# Advancing Sustainable Healthcare in Obstetric and Maternity Nursing: Nurses’ Knowledge, Awareness, and Clinical Practice—A Cross-Sectional Study

**DOI:** 10.3390/ijerph23060734

**Published:** 2026-05-30

**Authors:** Mirfat Mohamed Labib Elkashif, Doaa Mostafa Sheashaa, Mohamed Sayed Abdellatif, Darelglal Ahmed Gassmelseed, Shimaa Mohamed Mohamed Koabar, Sally Abd-Elrahman Mohamed

**Affiliations:** 1 Department of Nursing Sciences, College of Applied Medical Sciences in Wadi Aldawaser, Prince Sattam Bin Abdul-Aziz University, Wadi Aldawaser 18616, Saudi Arabia; 2 Obstetric and Gynecological Nursing, Faculty of Nursing, Delta University for Science & Technology, Gamasa 35712, Egypt; 3 Department of Psychology, College of Education in Al-Kharj, Prince Sattam Bin Abdulaziz University, Al-Kharj 11942, Saudi Arabia; 4 Public Health, Department of Public Health and Community Medicine, Faculty of Medicine, Tanta University, Tanta 31527, Egypt; shimaa_mohamed@med.tanta.edu.eg

**Keywords:** sustainable healthcare, obstetric nursing, maternity care, nurses’ knowledge, eco-conscious practices, sustainability training, environmental awareness

## Abstract

**Highlights:**

**Public health relevance—How does this work relate to a public health issue?**

- Healthcare services, especially maternity care, contribute to environmental pollution and greenhouse gas emissions, affecting population health.
- Integrating sustainability promotes a greener environment that benefits patients, healthcare workers, and communities.

**Public health significance—Why is this work of significance to public health?**

- Sustainable healthcare improves patient safety and quality of care while reducing environmental risks.
- It helps lower healthcare costs and reduces the burden on health systems through efficient resource use and waste reduction.

**Public health implications—What are the key implications or messages for practitioners, policy makers and/or researchers in public health?**

- Sustainability should be integrated into healthcare training and institutional policies to strengthen practice.
- Policy support is needed to implement green healthcare strategies that improve outcomes and environmental performance.

**Abstract:**

Background: Sustainable healthcare in obstetric and maternity nursing emphasizes the provision of high-quality, safe, and environmentally responsible care for women and newborns. Nurses’ knowledge, awareness, and clinical practices are central to the implementation of sustainable approaches, including efficient resource management, evidence-based interventions, and patient education. Evaluating these dimensions is essential for identifying gaps, informing targeted training, and supporting sustainable and effective maternal care aligned with global health goals. Accordingly, this study aimed to assess obstetric and maternity nurses’ knowledge, awareness, and clinical practices related to sustainable healthcare. Method: A cross-sectional study design was employed. A convenience sampling technique was used to recruit obstetric and maternity nurses working in the selected study settings during the data collection period. A total sample of 120 participants was targeted. The study was conducted at Al-Azhar University Hospital in New Damietta and selected Family Medicine Centers in Damietta Governorate, Egypt. Data were collected using a structured, self-administered questionnaire developed specifically for this study to assess eco-conscious nursing practices in obstetrics and gynecology units. The questionnaire included sections addressing demographic and professional characteristics, knowledge and awareness of sustainable healthcare, eco-conscious clinical practices in maternity settings, perceived barriers and institutional support, attitudes and advocacy toward environmental sustainability, procedure- and material-related environmental concerns, and energy and water conservation behaviors. Responses were measured using standardized 5-point Likert and frequency scales, with composite scores calculated to categorize levels of knowledge, practices, and attitudes toward sustainability; higher scores indicated greater knowledge, awareness, and engagement in sustainable practices. Results: Overall, among the 120 nurses, of whom 62 (51.7%) had reported having heard about sustainability and received training about it, whereas 58 (48.3%) had not. Most participants held a bachelor’s degree (*n* = 54, 45.0%), nearly half had more than 10 years of nursing experience (*n* = 58, 48.3%), and the largest proportion worked in delivery rooms (*n* = 53, 44.2%). Regarding knowledge, attitude, and practice, good knowledge was observed in 61 participants (50.8%), good practice in 46 participants (38.3%), and positive attitudes in 108 participants (90.0%). The findings also showed that trained nurses in obstetrics and gynecology units demonstrated significantly higher knowledge, more positive attitudes, and better eco-conscious practices compared to untrained nurses across all domains (*p* < 0.001). Conclusions: The study demonstrates that maternity nurses showed moderate to high awareness and positive attitudes toward sustainability, while environmentally sustainable practices were less consistently implemented, indicating a clear knowledge–attitude–practice gap. Nurses who received sustainability-related training consistently achieved significantly higher knowledge, attitude, and practice scores than untrained nurses.

## 1. Introduction

Sustainable healthcare has become a global priority as growing evidence demonstrates the substantial environmental impact of healthcare delivery and its direct and indirect consequences on human health. The concept of “hospital green practices” is therefore crucial, including 20–40% reductions in energy and water use, improved air quality, lower infection rates, and faster patient recovery. The WHO Green Hospital framework highlights the importance of renewable energy, non-toxic materials, climate-resilient infrastructure, and robust waste management in improving both staff safety and patient outcomes. Sustainability in healthcare is grounded in the broader definition: meeting present needs without compromising the ability of future generations to meet their own. In the clinical context, sustainable healthcare encompasses environmental, economic, and social dimensions. Sustainable healthcare aims to provide high-quality, patient-centered care while minimizing environmental harm and preserving resources for future generations. This approach aligns with the United Nations Sustainable Development Goals (SDGs), particularly SDG 3 (Good Health and Well-being), SDG 12 (Responsible Consumption and Production), and SDG 13 (Climate Action) [1,2,3,4,5,6,7].

Healthcare systems contribute considerably to environmental degradation through excessive energy consumption, greenhouse gas emissions, water use, and medical waste generation. Globally, healthcare activities account for approximately 4.4% of total carbon emissions, with hospitals representing some of the most resource-intensive institutions because of their extensive operational demands [1,2,3,4,5,6,7]. Clinical areas such as operating rooms, laboratories, and maternity units generate particularly high levels of waste and energy consumption. Inadequate waste segregation and reliance on disposable medical products further increase the environmental burden associated with healthcare delivery [1,2,3,4,5,6,7]. Additionally, anesthetic gases and environmentally hazardous disposal methods contribute substantially to greenhouse gas emissions and ecological pollution [1,2,3,4,5,6,7].

Within healthcare systems, sustainable hospital practices aim to reduce environmental impact while improving healthcare quality and operational efficiency. These practices include effective waste management, environmentally safe disposal systems, energy and water conservation, sustainable procurement policies, renewable energy integration, and the use of reusable medical supplies whenever clinically appropriate [1,2,3,4,5,6,7]. Green hospital initiatives have been associated with improved indoor air quality, reduced operational costs, safer healthcare environments, and enhanced patient and staff well-being [8,9,10].

Sustainability is particularly important in obstetric and maternity healthcare settings because maternity services require continuous clinical care, intensive resource utilization, and large volumes of medical supplies and waste production. Achieving SDG 3.1 by 2030 requires not only high-quality clinical care but also resilient and environmentally responsible healthcare systems [8,9,10].

Nurses play a central role in implementing sustainable healthcare practices because they are continuously involved in patient care, waste management, health education, and clinical decision-making. Obstetric and maternity nurses are particularly important because they work within highly resource-intensive environments and maintain continuous contact with women and newborns throughout pregnancy, labor, delivery, and postpartum care. Evidence suggests that reusable medical devices and delivery instruments may substantially reduce environmental impact compared with disposable alternatives [1,2,3,4,5,6,7]. Digital healthcare approaches, including telemedicine, may also help reduce unnecessary transportation-related emissions and improve healthcare accessibility for women living in remote areas [1,2,3,4,5,6,7]. Therefore, nurses’ sustainability-related knowledge, awareness, and clinical competencies are essential for translating sustainability principles into routine maternity care practice [11,12,13,14].

Despite increasing international attention toward sustainable healthcare, several barriers continue to limit the implementation of environmentally responsible clinical practices. Obstetric and maternity nurses frequently encounter inadequate sustainability. training, limited institutional support, insufficient financial resources, staff shortages, unclear organizational policies, and competing clinical priorities [1,2,3,4,5,6,7]. Organizational and systemic barriers may further hinder consistent implementation of sustainability initiatives within maternity healthcare settings [1,2,3,4,5,6,7].

Although many high-income countries have adopted comprehensive sustainability strategies within healthcare systems, low- and middle-income countries continue to face substantial structural and operational challenges. In Egypt, sustainability initiatives associated with Egypt Vision 2030 emphasize environmental protection, healthcare system resilience, responsible resource utilization, and improved waste management in alignment with the Sustainable Development Goals [1,2,3,4,5,6,7]. However, evidence regarding nurses’ sustainability-related knowledge and environmentally responsible maternity care practices within Egyptian healthcare settings remains limited.

Despite the growing global focus on sustainable healthcare, few studies have specifically examined obstetric and maternity nurses’ knowledge, awareness, attitudes, and eco-conscious clinical practices related to sustainable healthcare and hospital green practices in Egypt. In addition, limited evidence is available regarding the relationship between sustainability-related training and nurses’ implementation of environmentally responsible maternity care practices. Therefore, this study aimed to assess obstetric and maternity nurses’ knowledge, awareness, attitudes, and eco-conscious clinical practices related to sustainable healthcare and hospital green practices in selected maternity healthcare settings in Egypt.

## 2. Significance of the Study

Sustainable healthcare has become an essential global priority and is explicitly aligned with Egypt’s Vision 2030, which integrates sustainability as a key dimension of healthcare quality. In Egypt, a lower-middle-income country with high maternal and neonatal care demands, healthcare systems face ongoing challenges in balancing high-quality service delivery with environmental sustainability under conditions of resource limitation and increasing service pressure.

Obstetrics and gynecology nurses are central to implementing sustainable and environmentally responsible practices within maternity care settings. However, previous evidence indicates persistent gaps in nurses’ knowledge, awareness, attitudes, and clinical practices related to sustainability, including inadequate training in waste management, insufficient institutional support, and weak integration of green practices into routine clinical workflows.

Despite the global emphasis on sustainable healthcare, there is limited Egypt-specific evidence assessing obstetrics and gynecology nurses’ preparedness and actual clinical engagement with hospital green practices. This study therefore addresses this gap by explicitly examining nurses’ knowledge, awareness, attitudes, and clinical practices regarding sustainable healthcare in Egyptian maternity settings.

The study contributes novel, context-specific evidence that may support the development of targeted educational interventions, strengthen clinical practice, improve cost-effectiveness, and enhance the integration of sustainable healthcare principles into maternal and neonatal services in alignment with national and global sustainability goals.

## 3. Methods

### 3.1. Study Design

This study employed a cross-sectional design to assess obstetric and maternity nurses’ knowledge, awareness, attitudes, and clinical practices related to sustainable healthcare. This design was selected as it is appropriate for exploring current levels of sustainability-related competencies and practices at a single point in time.

### 3.2. Study Setting

The study was conducted in two types of healthcare settings in Damietta Governorate, Egypt: Al-Azhar University Hospital in New Damietta and selected Family Medicine Centers.

Al-Azhar University Hospital is a governmental teaching hospital providing comprehensive maternity services, including antenatal care, labor and delivery, postnatal care, and cesarean section services. The selected Family Medicine Centers provide primary maternal and reproductive healthcare services, including antenatal and postnatal follow-up and health education.

These settings were purposively selected to capture variability in sustainability-related nursing practices across both tertiary hospital-based maternity care and primary healthcare services. This allowed for a broader understanding of eco-conscious nursing practices within different levels of maternal healthcare delivery. The study was conducted over a three-month period from January to March 2026.

### 3.3. Sampling

A non-probability convenience sampling technique was used to recruit eligible obstetric and maternity nurses who were available in the selected settings during the data collection period. This approach was adopted due to logistical feasibility and the exploratory nature of the study; however, it may introduce a risk of selection bias, which is acknowledged as a study limitation.

### 3.4. Sample Size

A total sample of 120 nurses was targeted. This number was determined based on feasibility considerations, the availability of eligible participants in the selected settings, and the exploratory objective of the study rather than a formal power calculation.

All eligible nurses working in maternity and gynecology-related units at Al-Azhar University Hospital, as well as those providing maternal and reproductive healthcare services in the selected Family Medicine Centers, were invited to participate. This inclusive approach aimed to enhance the representation of nurses involved in both hospital-based and primary maternal care.

### 3.5. Inclusion Criteria

- Registered nurses working in maternity-related units (e.g., labor room, obstetrics and gynecology wards, or family medicine centers).
- Nurses with at least one year of clinical experience in maternity or obstetric care to ensure adequate exposure to clinical workflows and sustainability-related practices.
- Nurses who agreed to participate voluntarily in the study.

### 3.6. Exclusion Criteria

- Nurses on leave during the data collection period.
- Nurses in purely administrative positions without direct clinical involvement in maternity care, as the study focused on clinical sustainability practices.
- Nurses with less than one year of clinical experience in obstetric or maternity care.

### 3.7. Data Collection Procedure

Ethical approval was obtained from the Research Ethics Committee of the Faculty of Medicine, Al-Azhar University–Damietta prior to data collection.

Data were collected using a structured, self-administered questionnaire (not an interview-based tool), completed by participants under the supervision of the researcher to ensure clarity and completeness. This clarification addresses the distinction between interviews and questionnaire administration.

Eligible nurses working in antenatal, labor and delivery, postnatal wards, and operating rooms were identified according to the inclusion criteria. Participation was voluntary; oral informed consent was obtained from all participants. Confidentiality, anonymity, and the right to withdraw at any time were ensured.

The questionnaire was completed individually in a quiet area within the healthcare facilities during breaks to minimize disruption. Each participant required approximately 20–25 min to complete the tool, with the researcher available for clarification when needed.

### 3.8. Study Instrument

- Data was collected using a researcher-developed structured questionnaire designed after an extensive literature review and consultation with experts in nursing education and sustainable healthcare practice.
- The questionnaire was developed to assess eco-conscious nursing practices in obstetric and maternity settings and was informed by relevant literature on sustainability in healthcare [1,2,3,4,5,6,7]. The full English version of the tool is provided in Supplementary File S1.

The questionnaire was self-administered and completed anonymously by the participants during their work shifts. Participants required approximately 20–25 min to complete the questionnaire. Self-administered questionnaires were selected to ensure anonymity, reduce interviewer bias, and encourage honest responses regarding environmentally sustainable practices and attitudes. The instrument consisted of nine sections:

- Section S1: Demographic Characteristics: This section includes age, marital status, educational level, years of experience, and work unit.
- Section S2: Perceived Knowledge and Awareness of Sustainability (10 items): This section assessed participants’ knowledge and awareness of sustainability concepts in healthcare settings using a 5-point Likert scale ranging from 1 (strongly disagree) to 5 (strongly agree).
- Section S3: Eco-conscious Clinical Practices (17 items): This section measured the frequency of eco-friendly clinical behaviors using a 5-point frequency scale: 1 = Never, 2 = Rarely, 3 = Sometimes, 4 = Often, 5 = Always.
- Section S4: Barriers to Sustainability Practices (6 items): This section assessed perceived barriers hindering the implementation of sustainability practices using a 5-point Likert scale ranging from 1 (strongly disagree) to 5 (strongly agree).
- Section S5: Perceived Institutional and System-related Barriers (6 items): This section examined organizational and institutional obstacles affecting the adoption of sustainable practices using a 5-point Likert scale (1 = strongly disagree to 5 = strongly agree).
- Section S6: Attitudes and Advocacy Toward Sustainability (10 items): This section evaluated participants’ attitudes, beliefs, and advocacy behaviors regarding sustainability using a 5-point Likert scale (1 = strongly disagree to 5 = strongly agree).
- Section S7: Perceived Environmental and Procedural Concerns (5 items): This section assessed concerns related to environmental impact and clinical procedures in relation to sustainability using a 5-point Likert scale (1 = strongly disagree to 5 = strongly agree).
- Section S8: Material and Procedural Sustainability Practices (7 items): This section measured engagement in sustainable material use and clinical procedures using a 5-point Likert scale (1 = strongly disagree to 5 = strongly agree).
- Section S9: Energy and Water Conservation Practices (5 items): This section assessed practices related to energy and water conservation using a 5-point Likert scale (1 = strongly disagree to 5 = strongly agree).

The questionnaire constructs were grouped into the main study domains as follows:

- Knowledge domain: Section S2;
- Attitude domain: Sections S5–S7;
- Practice domain: Sections S3, S8 and S9.

### 3.9. Scoring System

Perceived knowledge and awareness scores (10 items): scored on a 5-point Likert scale (1–5) from strongly disagree to strongly agree, total score 10–50; categorized as poor (<50%), fair (50–75%), and good (>75%).

Attitudes (21 items across relevant sections): scored on a 5-point Likert scale from strongly disagree to strongly agree; total score 21–105, categorized as negative (<50%) and positive (≥50%).

Practice (29 items): All items measured sustainability-related practices, with scoring unified on a 5-point scale: (composite behavioral practice scale using heterogeneous Likert formats).

- Section S3: Frequency scale (behavioral occurrence) (1 = never to 5 = always).
- Sections S8 and S9: Agreement scale (behavioral endorsement) (1 = strongly disagree to 5 = strongly agree).

All items were coded so that higher scores consistently reflected higher levels of sustainable practice. The total practice score ranged from 27 to 145.

Higher scores indicated greater knowledge, more positive attitudes toward sustainable healthcare, and better self-reported eco-conscious clinical practices.

### 3.10. Validity and Reliability

The validity and reliability of the study instrument were rigorously assessed to ensure the accuracy, clarity, and consistency of the collected data. The initial version of the questionnaire was developed based on an extensive review of recent literature and relevant theoretical frameworks related to sustainable healthcare practices in nursing.

Content validity was established by a panel of five experts in maternal and women’s health nursing, nursing education, sustainable healthcare, and research methodology. The experts evaluated each item for clarity, relevance, simplicity, and alignment with the study objectives. The Content Validity Index (CVI) for the overall scale was high (0.98), indicating excellent content validity.

Face validity was assessed through a pilot study conducted with 12 nurses from a setting similar to the study population who were not included in the final sample. Participants provided feedback regarding item clarity, cultural appropriateness, comprehensibility, and ease of completion. Based on this feedback and expert recommendations, minor modifications were made to improve clarity and readability. Specifically, two items were removed, and three items were revised, resulting in a final version of 74 items.

The reliability of the instrument was assessed using Cronbach’s alpha coefficient to determine internal consistency. The Cronbach’s alpha coefficients were 0.74 for the knowledge domain, 0.81 for the attitude domain, and 0.79 for the practice domain, while the overall questionnaire reliability coefficient was 0.708, indicating acceptable internal consistency for exploratory research.

### 3.11. Pilot Study

A pilot study was carried out on (10%) 12 nurses to test the objectivity and applicability of the research tools and the feasibility of the research process. Data obtained from the pilot study were excluded from the final analysis.

The study achieved a 100% response rate, as all distributed questionnaires were completed and returned during the data collection period.

- *Ethical Approval:* Written informed consent was obtained from participants. This study was conducted in accordance with the ethical guidelines of the Institutional Review Board (IRB), Damietta Faculty of Medicine, Al Azhar University, and the Helsinki Declaration. DFM-IRB 00012367-25-12-008.

### 3.12. Statistical Analysis

The statistical analysis of the data was performed using IBM SPSS Statistics version 20.0 (IBM Corp: Armonk, NY, USA, released 2011). Categorical variables were summarized as frequencies and percentages, and associations were assessed using the Chi-square test or Monte Carlo simulation when appropriate. Monte Carlo corrected tests were used when expected cell counts were small or when Chi-square assumptions were violated. For continuous variables, normality was assessed using the Kolmogorov–Smirnov test, and data were presented as range (minimum–maximum), mean, and standard deviation. Spearman correlation analysis was conducted to assess relationships between nurses’ knowledge, attitude, and practice scores. Multiple linear regression analysis was performed to identify independent predictors of knowledge, attitude, and practice scores, adjusting for sociodemographic variables (age, marital status, education level, years of nursing experience, ward, and receipt of training). Statistical significance was set at *p* < 0.05.

## 4. Results

As presented in Table 1, a total of 120 nurses were included in the analysis. Slightly more than half of the participants reported previous exposure to sustainability-related training (*n* = 62, 51.7%), while 58 (48.3%) had not received such training. In this study, training refers to any formal or informal educational exposure (e.g., workshops, institutional sessions, or short courses) related to sustainable healthcare practices; however, the content and duration were not standardized across participants.

Most participants were married (51.7%) and held a bachelor’s degree (45.0%). Nearly half had more than 10 years of experience (48.3%), and the largest proportion worked in delivery rooms (44.2%). Slightly more than half (51.7%) reported prior awareness of sustainability in healthcare.

Regarding the main outcomes, half of the participants demonstrated good knowledge (50.8%), while only 38.3% showed good practice levels. In contrast, the majority (90.0%) demonstrated positive attitudes toward sustainable healthcare. Significant differences between trained and untrained nurses were observed for age, education level, years of experience, clinical ward, awareness of sustainability, and all outcome variables (knowledge, attitude, and practice) (*p* ≤ 0.001 for most comparisons). No significant difference was observed for marital status (*p* = 0.240). Overall, trained nurses consistently demonstrated higher knowledge, more favorable attitudes, and better practices compared with untrained nurses.

The mean knowledge, practice, and attitude scores were 39.25 ± 7.44, 103.58 ± 6.05, and 75.8 ± 15.6, respectively, indicating moderate to high levels across the three domains.

As presented in Table 2, multiple regression analysis revealed that receipt of sustainability training was the most consistent and strongest predictor across all outcome variables, showing statistically significant associations with knowledge (B = −14.333, *p* < 0.001), practice (B = −10.023, *p* < 0.001), and attitude scores (B = −28.606, *p* < 0.001). Ward assignment also significantly predicted both knowledge and practice scores, while years of nursing/maternity experience significantly predicted attitude and practice scores.

The regression models explained 93.5% of the variance in knowledge scores (R^2^ = 0.935, Adjusted R^2^ = 0.934, *p* < 0.001), 69.0% of the variance in practice scores (R^2^ = 0.690, Adjusted R^2^ = 0.688, *p* < 0.001), and 84.0% of the variance in attitude scores (R^2^ = 0.840, Adjusted R^2^ = 0.838, *p* < 0.001), indicating strong explanatory power of the models. Overall, the model indicates that exposure to training and clinical setting are key determinants of nurses’ eco-conscious knowledge, attitudes, and practices.

Spearman correlation analysis revealed a moderate positive correlation between knowledge and attitude scores (ρ = 0.478, *p* < 0.001), indicating that higher knowledge levels are associated with more positive attitudes toward sustainable practices.

As presented in Table 3, participants overall demonstrated moderate to high levels of sustainability awareness. The highest levels of agreement were observed for understanding environmental impacts of clinical practices, proper waste segregation, and recognition of the importance of sustainability training.

Trained nurses consistently demonstrated significantly higher awareness across most items compared with untrained nurses (*p* < 0.001 for most comparisons), indicating a strong association between training exposure and sustainability knowledge. Only a few items showed no statistically significant differences.

As shown in Table 4 at the organizational level, cost, staff engagement, and workload pressures were the most frequently reported barriers. Time constraints and procurement limitations also influenced the implementation of sustainable practices.

Trained nurses showed significantly higher awareness of institutional and policy-related barriers compared with untrained nurses (*p* ≤ 0.001), suggesting that training enhances critical awareness of system-level challenges.

Participants also demonstrated generally high levels of professional engagement in sustainability-related advocacy. Many nurses reported encouraging colleagues, discussing environmental health with patients, and recognizing their professional responsibility in promoting sustainability.

Participants demonstrated generally positive attitudes toward environmentally sustainable procedural practices in maternity care settings. High levels of agreement were observed regarding concerns about the environmental impact of anesthetic gases, appropriate medical waste segregation, and the potential replacement of single-use plastic drapes with reusable sterilizable cloth alternatives.

Trained nurses showed significantly higher engagement across all advocacy-related items (*p* < 0.001), indicating that training positively influences leadership behavior and environmental responsibility in clinical practice.

Notably, all participants reported awareness of the disposal methods for fetal monitoring straps and indicated the use of steam sterilization (autoclaving) more frequently than chemical sterilization. These findings may reflect standardized infection-control and sterilization protocols within the participating institutions rather than differences attributable to sustainability training.

As presented in Table 5, participants reported variable but generally moderate-to-high engagement in environmentally sustainable practices. The highest adherence was observed in core safety-related behaviors such as waste segregation, equipment shutdown after use, and supply management.

However, practices requiring behavioral change (e.g., reducing single-use materials and energy conservation behaviors) showed lower consistency.

Additionally, participants demonstrated generally high awareness regarding environmental sustainability issues related to material management and energy conservation within maternity settings. The highest levels of agreement were observed for awareness of the environmental impact of anesthesia gases, proper management of reusable materials, and recognition of the environmentally harmful effects of single-use plastics used in delivery kits and trays. Across all domains, trained nurses consistently demonstrated higher engagement and awareness (*p* < 0.001), highlighting the impact of sustainability training on clinical behavior.

**Figure 1:**
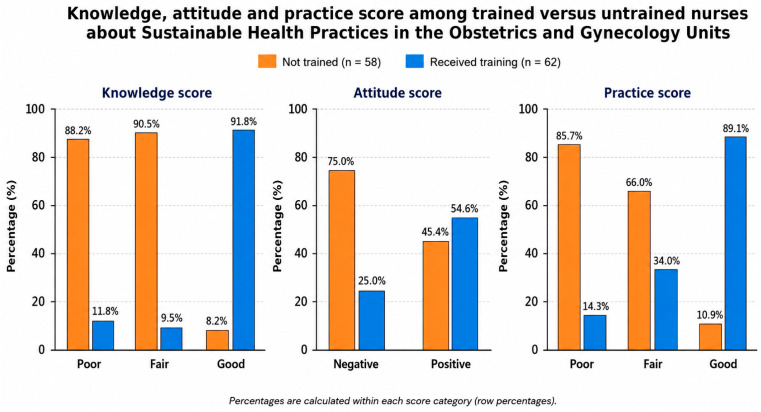
Knowledge, attitude and practice scores among trained versus untrained nurses.

Figure 1 illustrates a clear positive association between sustainability-related training and improved outcomes. Trained nurses demonstrated substantially higher levels of good knowledge, more favorable attitudes, and better clinical practices compared to untrained nurses.

Overall, trained nurses demonstrated significantly higher adherence to sustainable practices across most items (*p* < 0.001), particularly in material reuse, waste management, and energy conservation. Some routine hygiene-related practices showed no significant difference between groups.

As presented in Table 6, participants identified multiple barriers to implementing sustainable practices, with the most prominent being lack of consistency among staff and insufficient availability of sustainable alternatives.

Time constraints, limited managerial support, and concerns regarding infection control were also frequently reported. The cost of sustainable materials was universally recognized as a major barrier.

Training status influenced perceptions of barriers, with trained nurses generally reporting greater awareness of systemic and organizational constraints (*p* < 0.001 for several items), while no significant differences were observed for infrastructure-related barriers.

Overall, training was strongly associated with improved sustainability performance across all domains, emphasizing its critical role in enhancing environmentally responsible nursing practice in obstetric and maternity settings.

## 5. Discussion

In recent years, environmental sustainability in obstetrics and gynecology has received increasing global attention. The healthcare sector contributes approximately 4–10% of global greenhouse gas emissions. Given that obstetrics and gynecology nurses provide a wide range of high-volume services—including maternity, prenatal, pre-pregnancy, and reproductive care—implementing effective mitigation strategies in this specialty can significantly reduce the overall environmental footprint of healthcare systems.

Green hospital initiatives focus on strengthening waste management systems to reduce environmental harm through minimizing, segregating, and recycling medical waste. Additionally, the adoption of energy-efficient technologies can reduce energy consumption while offering long-term economic benefits [15].

The findings of the present study revealed that the mean age of participants was 36.52 ± 9.12 years, with nearly half being married. Approximately half held a bachelor’s degree, had prior awareness of sustainability concepts, had received related training, and had more than 10 years of nursing experience. The largest proportion worked in delivery rooms, reflecting the concentration of maternity care services. These findings are partially consistent with Allari et al. (2025), who reported a slightly younger workforce with a mean age of 34.33 ± 8.24 years, but limited formal training in green waste management practices [5]. Similar findings were also reported by Kebary et al. (2025), where many nurses had moderate clinical experience but lacked sustainability-related training [16]. Mohamed et al. (2025) further highlighted inadequate environmental infrastructure in maternal and child healthcare settings and noted that more than half of nurses resided in rural areas, reflecting broader contextual and system-level limitations in implementing sustainable healthcare practices [17].

The present study demonstrated that nurses working in obstetrics and gynecology units generally had moderate to high levels of sustainability-related knowledge. This level of awareness appears to be supported by prior exposure to sustainability concepts, participation in training activities, accumulated clinical experience, and frequent engagement in high-volume maternity care settings such as delivery rooms. Participants showed good understanding of environmentally responsible care, eco-friendly products, and waste classification. However, lower knowledge levels were observed regarding institutional sustainability initiatives, childbirth-related waste management protocols, and pharmaceutical waste handling. These findings suggest that although general awareness of sustainability is increasing, domain-specific operational and institution-specific knowledge remains insufficient, reflecting gaps in targeted education and clinical reinforcement.

Trained nurses consistently demonstrated higher knowledge, more favorable attitudes, and better practices compared with untrained nurses. This indicates the effect of education and training on increasing nurses’ awareness of sustainability. These findings are consistent with Araby et al. (2025), who reported significant improvements in nurses’ knowledge following structured educational interventions (*p* < 0.001) [3]. Similarly, Luque-Alcaraz et al. (2024) identified insufficient sustainability knowledge as a major barrier to implementing environmentally responsible practices in obstetric settings [18]. Additional studies by Hamid and Ibrahim (2025), Hamed et al. (2025), Topcu and Kiraz (2025), and Mohamed et al. (2025) further emphasized the effectiveness of structured training in improving sustainability-related knowledge and behaviors among nurses [17,18,19,20,21].

The broader literature also supports the growing role of nurses in promoting sustainability within healthcare systems. Algabar (2023), Dossey et al. (2019), and Álvarez-Nieto et al. (2022) emphasized that nurses contribute substantially to integrating sustainability principles into clinical practice and achieving Sustainable Development Goals (SDGs), particularly in resource-limited settings, attributing this to professional commitment to prevention and health promotion [1,22,23]. In contrast, Ovais (2023) reported low sustainability consciousness among nursing students, attributing this to limited engagement in research and insufficient institutional support [24]. Similarly, Taie (2022) found inadequate sustainability knowledge in perioperative settings due to the absence of integrated sustainability frameworks in care delivery systems [25].

Mixed findings have also been reported internationally. Allari et al. (2025) identified high sustainability consciousness among healthcare professionals in Amman, where social media was the main source of sustainability information, despite weak environmental behaviors and limited awareness of the Sustainable Development Goals (SDGs) [5,8,26,27]. Limited awareness of SDGs has been consistently linked to insufficient curricular integration, competing clinical priorities, and reliance on informal information sources such as social media, as reported across studies from Jordan, China, Pakistan, Egypt, Saudi Arabia, and the United States [5,28,29,30]. Similarly, Leal Filho et al. (2020) emphasized that weak institutional commitment and cultural constraints contribute to uneven sustainability engagement in healthcare systems [30]. The World Health Organization (WHO, 2023) therefore recommends integrating sustainability- and SDG-related content into health education and continuous professional development programs to strengthen sustainability literacy among healthcare professionals [9]. However, despite these recommendations, a persistent gap between intention and actual behavior remains evident. This intention–behavior gap has been widely documented and is largely influenced by cultural expectations, institutional constraints, and organizational readiness, as reported by Topcu and Kiraz (2025) and others [21,31,32,33]. Similarly, Huang et al. (2024) found that although healthcare workers demonstrated high concern for climate change (>90%), many lacked confidence in communicating its health implications, despite strong support for public education initiatives [8].

Regarding sustainability behaviors, the current study demonstrated generally positive engagement in environmentally responsible practices, particularly in advocacy- and leadership-related activities. Many participants reported encouraging colleagues, educating patients regarding environmental health, and supporting institutional green initiatives. Trained nurses consistently demonstrated stronger sustainability behaviors and greater professional commitment. These findings align with Algabar (2023), Sorour and Elkholy (2021), and Abd-Elmonem et al. (2022), who reported moderate to high sustainability-related behaviors among nurse managers and nurses, influenced by experience and participation in sustainability-focused training, particularly when structured training programs were available [1,34,35]. Similar conclusions were also reported by Fleiszer et al. (2016) and Salmela et al. (2016), emphasizing the role of leadership and organizational support in promoting sustainable healthcare practices, whereas Leppänen et al. (2022) highlighted that limited economic and ecological competencies may reduce nurses’ perceived ability to influence sustainability-related decisions [36,37,38].

Despite these positive findings, some environmental practices such as recycling and waste segregation remained suboptimal. Similar limitations were reported by Allari et al. (2025) and other studies [5]. This gap is reinforced by infrastructural and societal challenges in Jordan, including low public awareness, unclear responsibilities, and limited engagement in recycling behaviors, and reflects broader systemic and contextual barriers hindering sustainability practices [5,39,40,41,42]. These findings indicate that positive attitudes toward sustainability may not necessarily translate into consistent environmental behaviors without adequate institutional support and operational resources, as sustainability behaviors are shaped by a complex interaction of individual, organizational, sociocultural, and political factors [26,43,44,45,46].

Cultural and organizational factors may also influence sustainability engagement. Tam (2025) highlighted that pro-environmental behaviors are shaped by institutional expectations, cultural norms, and perceptions of responsibility [47]. In some healthcare systems, such as many Middle Eastern healthcare systems, environmental responsibility may be viewed primarily as a leadership obligation, limiting individual engagement despite positive attitudes [47,48,49]. Similarly, Vighnesh et al. (2024) highlighted these structural constraints, culturally embedded expectations and social norms hinder the translation of positive environmental values into sustained action [48]. These dynamics help explain why nurses in Jordan show stronger endorsement of social and economic sustainability (e.g., human rights, equity, and poverty reduction) compared with environmental behaviors. In contrast, weaker agreement with environmental behaviors—such as waste reduction or purchasing second-hand goods—may reflect prevailing cultural norms, limited institutional support, or insufficient emphasis on environmental sustainability within healthcare settings. Na’amneh and Al Husban (2012) further demonstrated that stigmatization of second-hand consumption in Jordan may suppress certain environmentally responsible behaviors despite their sustainability benefits, indicating that environmental considerations may be outweighed by cultural norms related to identity, status, and social acceptability [49]. Addressing these contextual barriers is therefore essential to promote meaningful behavioral change [48,49]. Leadership therefore remains essential in promoting sustainable healthcare environments. Rawson et al. (2025), Saleem et al. (2025), and the Centre for Sustainable Healthcare emphasized the importance of sustainability-oriented leadership and educational initiatives in motivating staff to act sustainably, thus strengthening environmental stewardship and healthcare quality [6,33].

The current findings further demonstrated that nurses who had received formal training showed substantially higher performance in sustainability-related practices. Similar improvements were reported by Araby et al. (2025), Mohammed et al. (2025), and Elgarf et al. (2023), who found significant increases in green practice scores following educational interventions [3,17,50,51]. The authors attributed these findings to the recent introduction of green practices in Egypt and anticipated further growth as awareness increases and sustainability becomes more fully integrated into obstetric and gynecologic services [50,51]. These studies emphasized that the use of diverse educational programs and visual materials, including PowerPoint presentations, video materials, illustrated booklets, and continuous professional development can improve awareness and address practice deficiencies.

The present study also identified several important barriers to implementing green healthcare practices in maternity settings. The most prominent barriers included inconsistent sustainability engagement among staff, time constraints during emergencies, limited availability of sustainable alternatives, inadequate managerial support, concerns about infection control with reusable products, low colleague “buy-in,” competing workload demands, procurement policies do not prioritize environmentally friendly options and high costs of environmentally friendly products. These findings align with Allari et al. (2025), Kebary et al. (2025), and Hassan et al. (2024), who similarly identified financial, infrastructural, administrative, governmental support and human-related challenges as major barriers to green healthcare implementation [5,16,52]. Comparable findings were observed in Egypt by Mohammed et al. (2025), indicating similar organizational and policy-related barriers across different healthcare systems [50]. Allari et al. attributed these challenges to the absence of dedicated monitoring teams, irregular follow-up, limited opportunities for self-development, gaps in knowledge regarding environmentally sound waste management, and a lack of awareness initiatives within obstetrics and gynecology departments. Additional constraints included the unavailability of appropriate waste-segregation containers, limited exposure to sustainable practices, unclear regulations and incentives, and low salaries contributing to job dissatisfaction [5]. Huang et al. (2024) additionally emphasized that limited organizational commitment and workforce challenges, including burnout and reduced professional engagement may negatively affect sustainability engagement among healthcare professionals and emphasize the importance of supporting staff well-being while promoting sustainability initiatives within healthcare systems [8]. Their study showed that healthcare workers often practiced more environmentally responsible behaviors outside the workplace than within clinical settings, creating tension between personal values and institutional practices. Participants also perceived organizational commitment to sustainability as limited, with most green initiatives driven by frontline staff rather than leadership [8].

A major contributor to perceived organizational barriers was limited awareness of environmental and newer climate-adaptation policies and climate preparedness initiatives, including the Health and Human Services (HHS) environmental goals, Environmental Sustainability Strategy, and Global Green and Healthy Hospitals (GGHH) membership programs. Awareness of these initiatives remained lower than that of more established healthcare campaigns, such as the “Choosing Wisely” initiative. This limited awareness may be related to the relatively recent introduction of environmental policies and the disruptions associated with the COVID-19 pandemic [50].

Correlation analysis in the present study demonstrated significant positive relationships between nurses’ knowledge, attitudes, and sustainability practices. Nurses with greater sustainability knowledge generally exhibited more favorable attitudes and stronger engagement in green practices. Algabar A.A. found significant positive correlations between nurses’ sustainability consciousness and both age and professional experience [1]. Younger nurses may demonstrate greater adaptability and responsiveness to sustainability messaging because of increased exposure to digital media and contemporary environmental campaigns, whereas more experienced nurses may exhibit stronger sustainability-oriented leadership and transformational roles [1]. Similar associations were reported by Alarri et al. (2025), Attia et al. (2025), Taie et al. (2025), Mohamed et al. (2025), and Dönmez and Yardımcı (2024), emphasizing that environmental knowledge is an important determinant of sustainable healthcare behaviors [5,17,25,53,54].

Significant associations were also observed between sustainability outcomes and several demographic and professional characteristics. Trained nurses, those with higher educational qualifications, greater clinical experience, and prior sustainability awareness demonstrated significantly higher knowledge, attitude, and practice scores. Nurses working in OB/GYN wards and delivery rooms were also more likely to report stronger sustainability engagement. Similar findings were reported by Allari et al. (2025), Mohamed et al. (2024), Magobe et al. (2025), Al-Bustanji et al. (2024), and Almukhaini et al. (2022), who noted that sustainability awareness and engagement tend to increase with professional experience, training exposure, leadership opportunities, and institutional involvement [5,11,55,56,57]. They noted that older professionals tend to show stronger sustainability attitudes and higher knowledge due to accumulated experience, greater exposure to institutional initiatives and a more developed understanding of long-term environmental consequences [11,55]. These findings highlight the importance of incorporating sustainability principles early in healthcare education and within continuing professional development. Algabar A.A. reported significant relationships between sustainable management behaviors and nurse managers’ age and income [1]. Older and better-compensated nurse managers were more likely to adopt sustainable management approaches. Adequate salaries were also associated with stronger responsibility, job satisfaction, and commitment to sustaining organizational practices [1].

Allari et al. (2025) reported that physicians demonstrated higher sustainability knowledge scores than nurses, which may be related to differences in training opportunities, professional authority, accumulated clinical experience, and organizational hierarchies within healthcare systems [5]. Similar findings were reported by Al-Bustanji et al. (2024) and Almukhaini et al. (2022), who noted that physicians often have greater access to information, professional autonomy, and institutional influence [56,57]. Addressing these hierarchical dynamics through interdisciplinary education and targeted sustainability training may help promote more equitable sustainability engagement across healthcare professions. Interventions should also be tailored according to professional groups and career stages to ensure that nurses and early-career staff receive adequate support in developing sustainability-related knowledge and skills [56,57].

A particularly notable finding was that more than half of participants were unfamiliar with the SDGs, consistent with findings from Saudi Arabia reported by Alonazi and Alkhateeb (2025) [58]. Additional studies by Algabar et al., Surzykiewicz et al. (2019), Wang et al. (2022), Peterlin et al. (2015), Iqbal and Ahmad (2021), and Gong et al. (2021) further emphasized the association between leadership, professional experience, and organizational culture in strengthening sustainability engagement, staff capacity development and promoting environmentally responsible behaviors among nurses [1,34,59,60,61,62,63,64].

Peterlin J. et al. (2015) emphasized that servant and sustainable leadership promote sustainability at the individual, organizational, and societal levels while addressing the needs of future generations [61]. Sorour A. and Elkholy S. (2021) also demonstrated positive associations between servant leadership, staff creativity, and sustainability behaviors [34]. In addition, Iqbal Q. and Ahmad N. (2021) described leadership frameworks that reinforce the pivotal role of sustainable leaders in strengthening sustainability engagement [62]. Furthermore, Gong Y. et al. (2021) found that nurses’ intentions to engage in green behaviors are enhanced in organizations characterized by ethical leadership environments [63]. Addressing these disparities requires interdisciplinary education, leadership opportunities, and tailored capacity-building interventions.

Overall, the findings of the present study highlight the important role of education, leadership, institutional support, and organizational readiness in promoting sustainable healthcare practices among maternity nurses.

### 5.1. Limitations of the Study

The cross-sectional design of the present study captures nurses’ perceptions and practices at a single point in time and therefore does not allow for the establishment of causal relationships between variables. In addition, the reliance on self-reported data may introduce recall bias and social desirability bias, which could have influenced participants’ responses toward more favorable reporting of knowledge and practices. The use of convenience sampling may also have contributed to selection bias, as participants who were more available or willing to participate may not fully represent the wider nursing population. Furthermore, the inclusion of a limited number of healthcare facilities restricts the generalizability of the findings to other settings or regions.

### 5.2. Implications for Practice

The findings of this study provide important baseline evidence to guide policy, education, and clinical practice aimed at strengthening environmental sustainability within Egyptian healthcare settings, particularly in maternity and obstetrics units. The results highlight the need for integrated institutional and educational strategies to enhance environmentally responsible behaviors among nurses, including waste segregation, energy and water conservation, and rational use of resources. Strengthening sustainability awareness through embedding SDG-related content into undergraduate curricula and continuous professional development programs is essential to bridge existing knowledge and practice gaps, especially among younger nurses and those with limited prior exposure.

At the policy level, hospital leadership and national health authorities should incorporate sustainability principles into accreditation standards, clinical protocols, and workforce development strategies to ensure alignment between organizational priorities and staff awareness. In addition, practical workplace support is required through the provision of appropriate infrastructure such as recycling systems, clearly designated waste streams, and environmentally friendly clinical supplies, alongside accessible evidence-based guidelines that facilitate consistent sustainable practice.

Educational and training initiatives should be designed to equip healthcare professionals with the knowledge, skills, and confidence needed to integrate planetary health principles into daily practice while also promoting eco-ethical leadership and interdisciplinary collaboration. Finally, fostering a culture of sustainability within healthcare organizations—where environmental responsibility becomes an integral component of routine clinical care alongside quality and patient safety—is essential. Empowering nurses as key change agents will be critical to advancing a more climate-resilient and environmentally responsible healthcare system.

## 6. Conclusions

Overall, the study demonstrates that maternity nurses exhibit moderate to high levels of sustainability awareness and generally positive attitudes toward environmental sustainability, while the actual implementation of sustainable practices remains inconsistent, indicating a clear knowledge–attitude–practice gap. Nurses who received sustainability-related training consistently demonstrated significantly higher knowledge, attitude, and practice scores compared with untrained nurses across all assessed domains (*p* < 0.001), highlighting the critical role of structured educational interventions in improving sustainability performance in clinical settings.

Training was strongly associated with improved engagement in key areas such as waste management, energy and water conservation, use of reusable materials, sustainable procurement, and environmental advocacy. In addition, a moderate positive correlation between knowledge and attitudes confirms that increased knowledge is associated with more favorable perceptions toward sustainability, which in turn supports better behavioral intention.

Despite these positive findings, several systemic and organizational barriers continue to limit full implementation of sustainable practices, including lack of staff consistency, the high cost of environmentally friendly alternatives, time constraints during emergencies, and limited availability of sustainable supplies. Notably, trained nurses demonstrated greater awareness of these barriers and showed stronger readiness to advocate for and lead sustainability initiatives within their workplaces.

### 6.1. Recommendations

Based on the findings of the present study, which demonstrated a clear knowledge–attitude–practice gap, the positive impact of training on sustainability performance, and the persistence of organizational and systemic barriers, the following recommendations are proposed to enhance sustainable, climate-resilient healthcare practice in maternity settings:

### 6.2. For Hospital Administration

- Implement structured and continuous training programs on sustainable healthcare practices, with emphasis on waste management, resource conservation, and environmental safety, as training was shown to significantly improve nurses’ knowledge, attitudes, and practices (*p* < 0.001).
- Strengthen institutional support by ensuring the availability of eco-friendly supplies, waste segregation systems, and energy- and water-saving infrastructure to reduce barriers identified in practice.
- Integrate sustainability competencies into job descriptions, clinical guidelines, and performance appraisal systems to ensure that sustainable behaviors become part of routine clinical expectations.
- Develop clear policies and operational frameworks that support green practices and address organizational barriers such as workload constraints and inconsistent staff engagement.
- Promote nurse engagement in sustainability initiatives by recognizing and rewarding environmentally responsible practices to reinforce positive behavioral change.

### 6.3. For Nurse Managers:

- Act as role models by integrating sustainability principles into daily clinical decision-making and unit management.
- Provide regular feedback, encouragement, and recognition to support nurses’ engagement in sustainable practices.
- Organize periodic in-service training sessions and short educational activities to continuously enhance sustainability awareness and practice.
- Facilitate access to updated information and practical tools related to environmental sustainability in healthcare.
- Encourage teamwork and shared responsibility to improve consistency in applying sustainable behaviors within clinical units.

### 6.4. For Nursing Education:

- Integrate environmental sustainability- and SDG-related content into undergraduate and postgraduate nursing curricula to address the observed gaps in knowledge and awareness.
- Provide students with practical exposure to environmental health activities within clinical training settings to strengthen real-world application of sustainability concepts.
- Promote a culture of sustainability through seminars, workshops, and community engagement activities focused on planetary health and environmental responsibility.

### 6.5. For Nursing Research:

- Conduct further studies to explore the long-term impact of sustainability training programs on clinical outcomes and healthcare quality indicators.
- Use qualitative research designs to gain deeper insight into organizational, cultural, and behavioral barriers influencing sustainable practice adoption.
- Investigate the relationship between sustainability practices, nurse empowerment, and patient outcomes to strengthen the evidence base for policy development.

## Figures and Tables

**Table 1:** Association Between Sociodemographic Characteristics and Receipt of Training among the studied participants (*n* = 120).

Variable	Category	Received Training			Test/ *p*-Value
		Yes 62 (51.7%)	No 58 (48.3%)	Total 120 (100%)	
Age (years) Mean ± SD	—	39.35 ± 9.22	33.48 ± 8.02	36.52 ± 9.12	t = 3.710
					*p* < 0.001 *
Marital status	Married	28 (45.2%)	34 (54.8%)	62 (51.7%)	MC = 2.850
	Single	22 (55.0%)	18 (45.0%)	40 (33.3%)	*p* = 0.240
	Widowed	12 (66.7%)	6 (33.3%)	18 (15.0%)	
Education level	Bachelor’s degree	25 (46.3%)	29 (53.7%)	54 (45.0%)	MC = 44.944
	Diploma	4 (12.9%)	27 (87.1%)	31 (25.8%)	*p* < 0.001 *
	Master’s degree	22 (91.7%)	2 (8.3%)	24 (20.0%)	
	Doctorate	11 (100.0%)	0 (0.0%)	11 (9.2%)	
Years of nursing experience	<1 year	1 (16.7%)	5 (83.3%)	6 (5.0%)	MC = 9.760
	1–5 years	6 (31.6%)	13 (68.4%)	19 (15.8%)	*p* = 0.021 *
	6–10 years	25 (67.6%)	12 (32.4%)	37 (30.8%)	
	>10 years	30 (51.7%)	28 (48.3%)	58 (48.3%)	
Ward	Delivery room	29 (54.7%)	24 (45.3%)	53 (44.2%)	MC = 14.702
	OB/GYN ward	24 (72.7%)	9 (27.3%)	33 (27.5%)	*p* = 0.001 *
	Outpatient	9 (26.5%)	25 (73.5%)	34 (28.3%)	
Heard about sustainability	Yes	62 (100.0%)	0 (0.0%)	62 (51.7%)	χ^2^ = 120.000
	No	0 (0.0%)	58 (100.0%)	58 (48.3%)	*p* < 0.001 *
Knowledge score	Poor	2 (11.8%)	15 (88.2%)	17 (14.2%)	MC = 120.000
	Fair	4 (9.5%)	38 (90.5%)	42 (35.0%)	*p* < 0.001 *
	Good	56 (91.8%)	5 (8.2%)	61 (50.8%)	
Attitude score	Negative	3 (25.0%)	9 (75.0%)	12 (10.0%)	χ^2^ = 10.401
	Positive	59 (54.6%)	49 (45.4%)	108 (90.0%)	*p* = 0.001 *
Practice score	Poor	3 (14.3%)	18 (85.7%)	21 (17.5%)	MC = 70.290
	Fair	18 (34.0%)	35 (66.0%)	53 (44.2%)	*p* < 0.001 *
	Good	41 (89.1%)	5 (10.9%)	46 (38.3%)	
Knowledge Score	Mean ± SD		39.25 ± 7.44		
Attitude score	Mean ± SD		75.8 ± 15.6		
Practice Score	Mean ± SD		103.5 ± 6.05		
Correlation analysis (Spearman’s rho)	Knowledge vs. Attitude score	—	—	ρ = 0.478	*p* < 0.001 *

t: Independent samples *t*-test; χ^2^: Chi-square test; MC: Monte Carlo exact test; ρ: Spearman’s rho correlation coefficient; * Statistically significant at *p* < 0.05.

**Table 2:** Multiple Linear Regression Analysis of Predictors of Nurses’ Knowledge, Attitude, and Practice Scores Regarding Sustainable Healthcare Practices in Obstetric and Maternity Settings (*p* < 0.05).

Outcome Variable	Significant Predictor	B	SE	β	t	*p*-Value	95% CI
Knowledge score	Ward	−0.951	0.192	−0.516	−4.958	<0.001 *	−1.335 to −0.567
	Source	0.731	0.108	0.766	6.777	<0.001 *	0.515 to 0.947
	Received training	−14.333	0.349	−0.967	−41.070	<0.001 *	−15.024 to −13.642
	Model statistics	R^2^ = 0.935	Adjusted R^2^ = 0.934	F = 1686.752		*p* < 0.001 *	
Attitude score	Years of maternity experience	3.242	1.078	0.407	3.007	0.004 *	1.082 to 5.401
	Received training	−28.606	1.151	−0.916	−24.844	<0.001 *	−30.886 to −26.326
	Model statistics	R^2^ = 0.840	Adjusted R^2^ = 0.838	F = 617.215		*p* < 0.001 *	
Practice score	Years of nursing experience	0.944	0.373	0.335	2.529	0.014 *	0.196 to 1.691
	Ward	−0.836	0.378	−0.294	−2.209	0.031 *	−1.593 to −0.078
	Received training	−10.023	0.618	−0.831	−16.215	<0.001 *	−11.247 to −8.799
	Model statistics	R^2^ = 0.690	Adjusted R^2^ = 0.688	F = 262.934		*p* < 0.001 *	

Training variable coded as: Yes = 1, No = 2/β = Standardized beta coefficient. R^2^ = coefficient of determination. CI = confidence interval. Only statistically significant predictors are presented (*p* < 0.05). * Statistically significant at *p* < 0.05.

**Table 3:** Perceived Knowledge and Awareness of Sustainability in Healthcare among the studied participants (*n* = 120).

Variable	Response	Received Training			Test/ *p*-Value
		Yes 62 (51.7%)	No 58 (48.3%)	Total 120 (100%)	
Q2_1 I am familiar with the concept of “sustainable healthcare” in the context of the maternity unit	Strongly Disagree	0 (0.0)	3 (100.0)	3 (2.5)	MC = 105.471
	Disagree	0 (0.0)	27 (100.0)	27 (22.5)	*p* < 0.001 *
	Neutral	1 (3.8)	25 (96.2)	26 (21.7)	
	Agree	24 (88.9)	3 (11.1)	27 (22.5)	
	Strongly Agree	37 (100.0)	0 (0.0)	37 (30.8)	
Q2_2”Sustainable healthcare” means providing care without compromising the environment for future generations	Disagree	0 (0.0)	2 (100.0)	2 (1.7)	MC = 99.326
	Neutral	0 (0.0)	19 (100.0)	19 (15.8)	*p* < 0.001 *
	Agree	6 (14.0)	37 (86.0)	43 (35.8)	
	Strongly Agree	56 (100.0)	0 (0.0)	56 (46.7)	
Q2_3 I understand the link between the use of certain chemicals in the maternity unit and environmental pollution	Disagree	0 (0.0)	1 (100.0)	1 (0.8)	MC = 2.904
	Neutral	1 (33.3)	2 (66.7)	3 (2.5)	*p* = 0.407
	Agree	18 (62.1)	11 (37.9)	29 (24.2)	
	Strongly Agree	43 (49.4)	44 (50.6)	87 (72.5)	
Q2_4 I am aware of the correct protocols for pharmaceutical waste disposal to prevent environmental contamination	Strongly Disagree	0 (0.0)	2 (100.0)	2 (1.7)	MC = 91.397
	Disagree	0 (0.0)	31 (100.0)	31 (25.8)	*p* < 0.001 *
	Neutral	10 (28.6)	25 (71.4)	35 (29.2)	
	Agree	31 (100.0)	0 (0.0)	31 (25.8)	
	Strongly Agree	21 (100.0)	0 (0.0)	21 (17.5)	
Q2_5 The majority of waste generated in my unit is general waste, not clinical waste	Disagree	0 (0.0)	1 (100.0)	1 (0.8)	MC = 6.797
	Neutral	2 (100.0)	0 (0.0)	2 (1.7)	*p* = 0.079
	Agree	15 (38.5)	24 (61.5)	39 (32.5)	
	Strongly Agree	45 (57.7)	33 (42.3)	78 (65.0)	
Q2_6 I am confident in my ability to identify products in the maternity unit that are environmentally friendly (e.g., biodegradable, recycled)	Disagree	0 (0.0)	11 (100.0)	11 (9.2)	MC = 106.720
	Neutral	3 (6.1)	46 (93.9)	49 (40.8)	*p* < 0.001 *
	Agree	1 (50.0)	1 (50.0)	2 (1.7)	
	Strongly Agree	58 (100.0)	0 (0.0)	58 (48.3)	
Q2_7 I know the correct disposal protocol for all types of clinical and non-clinical waste generated during a birth	Strongly Disagree	0 (0.0)	11 (100.0)	11 (9.2)	MC = 99.978
	Disagree	0 (0.0)	17 (100.0)	17 (14.2)	*p* < 0.001 *
	Neutral	6 (16.7)	30 (83.3)	36 (30.0)	
	Agree	35 (100.0)	0 (0.0)	35 (29.2)	
	Strongly Agree	21 (100.0)	0 (0.0)	21 (17.5)	
Q2_8 I am aware of my hospital’s current sustainability initiatives	Disagree	0 (0.0)	25 (100.0)	25 (20.8)	MC = 113.114
	Neutral	1 (3.0)	32 (97.0)	33 (27.5)	*p* < 0.001 *
	Agree	3 (75.0)	1 (25.0)	4 (3.3)	
	Strongly Agree	58 (100.0)	0 (0.0)	58 (48.3)	
Q2_9 Formal training on environmental best practices is essential for my role	Disagree	0 (0.0)	13 (100.0)	13 (10.8)	MC = 70.732
	Neutral	0 (0.0)	20 (100.0)	20 (16.7)	*p* < 0.001 *
	Agree	1 (8.3)	11 (91.7)	12 (10.0)	
	Strongly Agree	61 (81.3)	14 (18.7)	75 (62.5)	
Q2_10 I know how to correctly dispose of different types of waste generated in the maternity unit	Disagree	0 (0.0)	27 (100.0)	27 (22.5)	MC = 53.090
	Neutral	0 (0.0)	7 (100.0)	7 (5.8)	*p* < 0.001 *
	Agree	55 (69.6)	24 (30.4)	79 (65.8)	
	Strongly Agree	7 (100.0)	0 (0.0)	7 (5.8)	

MC: Monte Carlo test; *: Statistically significant (*p* < 0.05).

**Table 4:** Association Between Received Training and Nurses’ Eco-conscious Advocacy, Attitudes, and Engagement in Sustainability Practices (Q5–Q7) among the studied participants (*n* = 120).

Variable	Category	Received Training			Test/ *p*-Value
		Yes 62 (51.7%)	No 58 (48.3%)	Total 120 (100%)	
Q5_1 There is a lack of clear guidance from management on sustainable practices in my unit	Neutral	62 (51.7%)	58 (48.3%)	120 (100%)	—
Q5_2 Time constraints during busy shifts make it difficult to focus on waste segregation or energy saving	Strongly Disagree	49 (64.5%)	27 (35.5%)	76 (63.3%)	MC = 32.071
	Disagree	13 (65.0%)	7 (35.0%)	20 (16.7%)	*p* < 0.001 *
	Neutral	0 (0.0%)	24 (100.0%)	24 (20.0%)	
Q5_3 The hospital’s current procurement policies do not prioritize environmentally friendly products	Neutral	62 (62.0%)	38 (38.0%)	100 (83.3%)	χ^2^ = 25.655
	Agree	0 (0.0%)	20 (100.0%)	20 (16.7%)	*p* < 0.001 *
Q5_4 There is a “buy-in” problem among my colleagues; many are not interested in sustainability efforts	Agree	0 (0.0%)	9 (100.0%)	9 (7.5%)	χ^2^ = 10.401
	Strongly Agree	62 (55.9%)	49 (44.1%)	111 (92.5%)	*p* = 0.001 *
Q5_5 I have received formal training on waste management specific to the operating room or delivery ward	Strongly Disagree	1 (7.1%)	13 (92.9%)	14 (11.7%)	MC = 112.945
	Disagree	0 (0.0%)	44 (100.0%)	44 (36.7%)	*p* < 0.001 *
	Agree	5 (83.3%)	1 (16.7%)	6 (5.0%)	
	Strongly Agree	56 (100.0%)	0 (0.0%)	56 (46.7%)	
Q5_6 The cost of sustainable alternatives is perceived as a major barrier by the administration	Agree	62 (51.7%)	58 (48.3%)	120 (100%)	—
Q6_1 I actively encourage my colleagues	Strongly Disagree	1 (5.3%)	18 (94.7%)	19 (15.8%)	MC = 83.972
	Disagree	0 (0.0%)	30 (100%)	30 (25.0%)	*p* < 0.001 *
	Agree	13 (72.2%)	5 (27.8%)	18 (15.0%)	
	Strongly Agree	21 (95.5%)	1 (4.5%)	31 (25.8%)	
Q6_2 I discuss importance of environmental health	Strongly Disagree	27 (87.1%)	4 (12.9%)	14 (11.7%)	MC = 99.573
	Disagree	1 (7.1%)	13 (92.9%)	39 (32.5%)	*p* < 0.001 *
	Agree	0 (0.0%)	39 (100%)	14 (11.7%)	
	Strongly Agree	9 (64.3%)	5 (35.7%)	29 (24.2%)	
Q6_3 Nurses’ professional obligation to advocate	Strongly Disagree	23 (95.8%)	1 (4.2%)	21 (17.5%)	MC = 94.295
	Disagree	29 (100%)	0 (0.0%)	30 (25.0%)	*p* < 0.001 *
	Agree	1 (4.8%)	20 (95.2%)	20 (16.7%)	
	Strongly Agree	0 (0.0%)	30 (100%)	37 (30.8%)	
Q6_4 Participation in hospital-wide initiative	Strongly Disagree	13 (65.0%)	7 (35.0%)	14 (11.7%)	MC = 112.678
	Disagree	11 (91.7%)	1 (8.3%)	44 (36.7%)	*p* < 0.001 *
	Agree	37 (100%)	0 (0.0%)	6 (5.0%)	
	Strongly Agree	1 (7.1%)	13 (92.9%)	46 (38.3%)	
Q6_5 Willing to lead sustainability project	Strongly Disagree	1 (5.3%)	18 (94.7%)	19 (15.8%)	MC = 106.013
	Disagree	0 (0.0%)	36 (100%)	36 (30.0%)	*p* < 0.001 *
	Agree	7 (100%)	0 (0.0%)	7 (5.8%)	
	Strongly Agree	7 (63.6%)	4 (36.4%)	47 (39.2%)	
Q6_6 “Being green” improves outcomes	Strongly Disagree	47 (100%)	0 (0.0%)	24 (20.0%)	MC = 112.522
	Disagree	1 (4.2%)	23 (95.8%)	34 (28.3%)	*p* < 0.001*
	Agree	0 (0.0%)	34 (100%)	7 (5.8%)	
	Strongly Agree	7 (100%)	0 (0.0%)	44 (36.7%)	
Q6_7 Empowered to suggest process improvements	Strongly Disagree	10 (90.9%)	1 (9.1%)	16 (13.3%)	MC = 85.278
	Disagree	44 (100%)	0 (0.0%)	38 (31.7%)	*p* < 0.001 *
	Agree	3 (18.8%)	13 (81.3%)	6 (5.0%)	
	Strongly Agree	0 (0.0%)	38 (100%)	60 (50.0%)	
Q6_8 Nurses as key players in sustainability	Strongly Disagree	5 (83.3%)	1 (16.7%)	14 (11.7%)	MC = 101.332
	Disagree	54 (90.0%)	6 (10.0%)	33 (27.5%)	*p* < 0.001 *
	Agree	1 (7.1%)	13 (92.9%)	15 (12.5%)	
	Strongly Agree	0 (0.0%)	33 (100%)	54 (45.0%)	
Q6_9 Advocating for healthy environment	Strongly Disagree	0 (0.0%)	4 (100%)	14 (11.7%)	MC = 91.243
	Disagree	7 (46.7%)	8 (53.3%)	31 (25.8%)	*p* < 0.001 *
	Agree	54 (100%)	0 (0.0%)	6 (5.0%)	
	Strongly Agree	1 (7.1%)	13 (92.9%)	62 (51.7%)	
Q6_10 Discuss environmentally friendly newborn care	Strongly Disagree	0 (0.0%)	31 (100%)	14 (11.7%)	MC = 74.117
	Disagree	0 (0.0%)	7 (100%)	25 (20.8%)	*p* < 0.001 *
	Agree	5 (83.3%)	1 (16.7%)	17 (14.2%)	
	Strongly Agree	56 (90.3%)	6 (9.7%)	64 (53.3%)	
Specific Procedural Concerns (Anesthesia, Instruments, Waste)					
Q7_1 The environmental impact of anesthetic gases (e.g., nitrous oxide) used for pain relief in labor is a concern for me	Neutral	0 (0.0)	18 (100.0)	18 (15.0)	MC = 27.205
	Agree	25 (50.0)	25 (50.0)	50 (41.7)	*p* < 0.001 *
	Strongly Agree	37 (71.2)	15 (28.8)	52 (43.3)	
Q7_2 Our unit effectively separates “red bag” regulated medical waste from standard general waste	Agree	62 (63.3)	36 (36.7)	98 (81.7)	χ^2^ = 28.797
	Strongly Agree	0 (0.0)	22 (100.0)	22 (18.3)	*p* < 0.001 *
Q7_3 I am aware of the specific disposal method for single-use fetal monitoring straps/sensors	Strongly Agree	62 (51.7)	58 (48.3)	120 (100.0)	—
Q7_4 We use steam sterilization (autoclave) of instruments more often than chemical sterilization	Strongly Agree	62 (51.7)	58 (48.3)	120 (100.0)	—
Q7_5 I believe single-use plastic drapes used during C-sections or deliveries could be replaced with sterilizable cloth ones	Neutral	0 (0.0)	25 (100.0)	25 (20.8)	MC = 33.956
	Agree	46 (66.7)	23 (33.3)	69 (57.5)	*p* < 0.001 *
	Strongly Agree	16 (61.5)	10 (38.5)	26 (21.7)	

χ^2^: Chi-square test. MC: Monte Carlo test; *: Statistically significant (*p*< 0.05).

**Table 5:** Eco-conscious Practices in Maternity Ward Procedures Among the Studied Participants (*n* = 120).

Variable	Response	Received Training			Test/ *p*-Value
		Yes 62 (51.7%)	No 58 (48.3%)	Total 120 (100%)	
Q3_1 I prioritize using cloth towels/gowns over disposable paper ones	Never	13 (34.2)	25 (65.8)	38 (31.7)	MC = 13.284
	Rarely	23 (47.9)	25 (52.1)	48 (40.0)	*p* = 0.001 *
	Sometimes	26 (76.5)	8 (23.5)	34 (28.3)	
Q3_2 I minimize the use of single-use plastic drapes and coverings	Rarely	18 (35.3)	33 (64.7)	51 (42.5)	χ^2^ = 9.521
	Sometimes	44 (63.8)	25 (36.2)	69 (57.5)	*p* = 0.002 *
Q3_3 I ensure all clean recyclable packaging is properly recycled	Never	62 (51.7)	58 (48.3)	120 (100.0)	—
Q3_4 I power down electronic equipment when not needed	Always	62 (51.7)	58 (48.3)	120 (100.0)	—
Q3_5 I opt for saline flushes in reusable vials instead of single-use	Never	62 (51.7)	58 (48.3)	120 (100.0)	—
Q3_6 I carefully manage supplies opened for each procedure	Always	62 (51.7)	58 (48.3)	120 (100.0)	—
Q3_7 I check expiration dates frequently	Always	62 (51.7)	58 (48.3)	120 (100.0)	—
Q3_8 I use environmentally friendly cleaning products	Often	46 (47.9)	50 (52.1)	96 (80.0)	χ^2^ = 2.703
	Always	16 (66.7)	8 (33.3)	24 (20.0)	*p* = 0.100
Q3_9 I turn off lights/equipment when not in use	Rarely	0 (0.0)	25 (100.0)	25 (20.8)	χ^2^ = 33.757
	Sometimes	62 (65.3)	33 (34.7)	95 (79.2)	*p* < 0.001 *
Q3_10 I minimize paper usage	Rarely	35 (51.5)	33 (48.5)	68 (56.7)	χ^2^ = 0.002
	Sometimes	27 (51.9)	25 (48.1)	52 (43.3)	*p* = 0.961
Q3_11 I actively participate in the unit’s recycling programs	Never	62 (51.7)	58 (48.3)	120 (100.0)	—
Q3_12 I choose reusable equipment/materials when clinically safe	Often	1 (3.8)	25 (96.2)	26 (21.7)	χ^2^ = 30.395
	Always	61 (64.9)	33 (35.1)	94 (78.3)	*p* < 0.001 *
Q3_13 I ensure proper segregation of general, clinical, and hazardous waste	Always	62 (51.7)	58 (48.3)	120 (100.0)	—
Q3_14 I use water conservation practices during procedures or cleaning	Rarely	0 (0.0)	15 (100.0)	15 (12.5)	MC = 25.246
	Sometimes	34 (70.8)	14 (29.2)	48 (40.0)	*p* < 0.001 *
	Often	28 (50.9)	27 (49.1)	55 (45.8)	
	Always	0 (0.0)	2 (100.0)	2 (1.7)	
Q3_15 I consider environmental impact when selecting medical supplies	Rarely	0 (0.0)	13 (100.0)	13 (10.8)	MC = 15.763
	Sometimes	16 (61.5)	10 (38.5)	26 (21.7)	*p* < 0.001 *
	Often	46 (56.8)	35 (43.2)	81 (67.5)	
Q3_16 I report inefficiencies in waste management or energy use	Always	62 (51.7)	58 (48.3)	120 (100.0)	—
Q3_17 I manage supply “prep” carefully to avoid unnecessary waste	Always	62 (51.7)	58 (48.3)	120 (100.0)	—
Material Management					
Q8_1 I am aware of the environmental burden associated with single-use plastics used in delivery kits/trays	Strongly Disagree	1 (7.1)	13 (92.9)	14 (11.7)	MC = 112.945
	Disagree	0 (0.0)	44 (100.0)	44 (36.7)	*p* < 0.001 *
	Agree	5 (83.3)	1 (16.7)	6 (5.0)	
	Strongly Agree	56 (100.0)	0 (0.0)	56 (46.7)	
Q8_2 Our unit over-packs procedure trays, leading to unnecessary waste of supplies	strongly Disagree	62 (51.7)	58 (48.3)	120 (100.0)	—
Q8_3 I feel our unit has effective systems for returning unopened, unused sterile supplies to stock	Strongly Agree	62 (51.7)	58 (48.3)	120 (100.0)	—
Q8_4 I use the minimum amount of antiseptic solution/disinfectant required for procedures to avoid chemical waste	Strongly Agree	62 (51.7)	58 (48.3)	120 (100.0)	—
Q8_5 I am comfortable suggesting the use of reusable cloth items over disposables	Strongly Disagree	1 (7.1)	13 (92.9)	14 (11.7)	MC = 112.945
	Disagree	0 (0.0)	44 (100.0)	44 (36.7)	*p* < 0.001 *
	Agree	5 (83.3)	1 (16.7)	6 (5.0)	
	Strongly Agree	56 (100.0)	0 (0.0)	56 (46.7)	
Q8_6 Anesthesia gases used during deliveries have a significant environmental impact	Neutral	1 (3.6)	27 (96.4)	28 (23.3)	MC = 39.289
	Agree	0 (0.0)	3 (100.0)	3 (2.5)	*p* < 0.001 *
	Strongly Agree	61 (68.5)	28 (31.5)	89 (74.2)	
Q8_7 I advocate for the use of less environmentally harmful pain management options during labor	Strongly Agree	62 (51.7)	58 (48.3)	120 (100.0)	—
Energy and Water Consumption in the Unit					
Q9_1I ensure non-critical monitors and medical devices are powered off when not in use	Neutral	1 (3.6)	27 (96.4)	28 (23.3)	MC = 39.289
	Agree	0 (0.0)	3 (100.0)	3 (2.5)	*p* < 0.001 *
	Strongly Agree	61 (68.5)	28 (31.5)	89 (74.2)	
Q9_2 Our maternity unit’s current lighting system is energy efficient	Neutral	15 (68.2)	7 (31.8)	22 (18.3)	χ^2^ = 2.942
	Agree	47 (48.0)	51 (52.0)	98 (81.7)	*p* = 0.086
Q9_3 I take steps to minimize running water during bathing, cleaning, or other procedures	Disagree	48 (56.5)	37 (43.5)	85 (70.8)	χ^2^ = 2.693
	Neutral	14 (40.0)	21 (60.0)	35 (29.2)	*p* = 0.101
Q9_4 The temperature in the maternity unit is often too high, leading to wasted energy	Neutral	62 (51.7)	58 (48.3)	120 (100.0)	—
Q9_5 I am aware of the specific energy consumption ‘hotspots’ in the maternity war_	agree	62 (51.7)	58 (48.3)	120 (100.0)	—

χ^2^: Chi-square test; MC: Monte Carlo test; *: Statistically significant (*p* < 0.05). Statistical tests were not computed for variables with constant responses across all participants.

**Table 6:** Association Between Received Training and Perceived Barriers to Sustainable Maternity Nursing Practice Among the Studied Participants (*n* = 120).

Variable	Response	Received Training			Test/ *p*-Value
		Yes 62 (51.7%)	No 58 (48.3%)	Total 120 (100%)	
Q4_1 Time constraints during emergencies prevent focusing on waste segregation or energy saving	Moderate Barrier	0 (0.0%)	22 (100.0%)	22 (18.3%)	MC = 28.804
	Major Barrier	16 (64.0%)	9 (36.0%)	25 (20.8%)	*p* < 0.001 *
	Significant Barrier	46 (63.0%)	27 (37.0%)	73 (60.8%)	
Q4_2 The layout of the L&D rooms makes proper waste segregation bins difficult to access	Not a Barrier	46 (47.9%)	50 (52.1%)	96 (80.0%)	χ^2^ = 2.703
	Minor Barrier	16 (66.7%)	8 (33.3%)	24 (20.0%)	*p* = 0.100
Q4_3 Lack of supportive management or clear policies on sustainability from hospital leadership	Not a Barrier	24 (96.0%)	1 (4.0%)	25 (20.8%)	MC = 101.095
	Minor Barrier	12 (92.3%)	1 (7.7%)	13 (10.8%)	*p* < 0.001 *
	Moderate Barrier	23 (100.0%)	0 (0.0%)	23 (19.2%)	
	Major Barrier	1 (3.7%)	26 (96.3%)	27 (22.5%)	
	Significant Barrier	2 (6.3%)	30 (93.8%)	32 (26.7%)	
Q4_4 Concerns that “green” products or reusable items may compromise infection control standards	Not a Barrier	38 (57.6%)	28 (42.4%)	66 (55.0%)	χ^2^ = 2.051
	Minor Barrier	24 (44.4%)	30 (55.6%)	54 (45.0%)	*p* = 0.152
Q4_5 Insufficient supply of sustainable alternatives to current single-use products	Major Barrier	16 (61.5%)	10 (38.5%)	26 (21.7%)	χ^2^ = 1.295
	Significant Barrier	46 (48.9%)	48 (51.1%)	94 (78.3%)	*p* = 0.255
Q4_6 Lack of consistency in sustainability efforts among all L&D staff members	Significant Barrier	62 (51.7%)	58 (48.3%)	120 (100%)	—

χ^2^: Chi-square test; MC: Monte Carlo test; *: Statistically significant (*p* < 0.05).

## Data Availability

The datasets used and analyzed during the current study are available from the corresponding author on reasonable request.

## Supplementary Materials

The following supporting information can be downloaded at: https://www.mdpi.com/article/10.3390/ijerph23060734/s1. File S1: The researcher-developed structured questionnaire.

## Abbreviations

SDG	Sustainable Developmental Goals
HHS	Health and Human Services
GGHH	Global Green and Healthy Hospitals
