# Supplementary material for: Advancing Sustainable Healthcare in Obstetric and Maternity Nursing: Nurses’ Knowledge, Awareness, and Clinical Practice—A Cross-Sectional Study
Source: Int J Environ Res Public Health. 2026 May 30;23(6):734. doi: 10.3390/ijerph23060734 (PMC13299454; doi:10.3390/ijerph23060734)
Supplement: Supplementary file 1 [file ijerph-23-00734-s001.zip › ijerph-4292205-supplementary.pdf]

Advancing Sustainable Healthcare in Obstetric and Maternity Nursing: Nurses' Knowledge, Awareness, and Clinical Practice A Cross-Sectional Study.

***This questionnaire is designed to evaluate current sustainable health practices and eco-conscious behaviors among nursing staff in a delivery unit. The goal is to identify existing strengths, knowledge gaps, and opportunities for improvement in environmentally responsible care. This survey can be adapted for research, quality improvement, or staff education purposes.

Instructions for respondents:

- Please answer each question honestly based on your knowledge and experience in this
- our responses will be kept confidential.
- The results will be used to improve our department's sustainability practices.

Section S1: Demographic Information:

Age: -			
Marital status:			
Highest Level of Education	Diploma (Associate Degree in Nursing)	Bachelor's Degree	
	Master's Degree	Doctorate	
	Other: [Specify]		
Years of Experience in Nursing	Less than 1 year	6–10 years	
	1–5 years	More than 10 years	
Years of Experience in Maternity	Less than 1 year	6–10 years	
	1–5 years	More than 10 years	
The ward you are working in	Delivery room	Obegyne ward	Out patient
Have you received any formal education or training on sustainable practices in obstetric care?	Yes,<br>If yes, please specify the type of training: ___		
	No		
Hearing about sustainability development	Yes, Where:		
	No,		

Section S2: Knowledge and Awareness of Sustainability in Healthcare:-

(General Sustainability Awareness and Knowledge)

Use a Likert scale: **1 = Strongly Disagree**, **2 = Disagree, 3 = Neutral, 4 = Agree, 5 = Strongly Agree**

No	Statement	1	2	3	4	5
1	I am familiar with the concept of "sustainable healthcare" in the context of the maternity unit					

2	Sustainable healthcare" means providing care without compromising the environment for future generations.					
3	I understand the link between the use of certain chemicals in the maternity unit and environmental pollution.					
4	I am aware of the correct protocols for pharmaceutical waste disposal to prevent environmental contamination.					
5	The majority of waste generated in my unit is general waste, not clinical waste.					
6	I am confident in my ability to identify products in the maternity unit that are environmentally friendly (e.g., biodegradable, recycled).					
7	I know the correct disposal protocol for all types of clinical and non-clinical waste generated during a birth.					
8	I am aware of my hospital's current sustainability initiatives.					
9	Formal training on environmental best practices is essential for my role.					
10	I know how to correctly dispose of different types of waste generated in the maternity unit.					

Section S3: Eco-conscious Practices in maternity ward Procedures (Eco-conscious Practices in Medical Procedures (Maternity Specific).

Use a frequency scale: **1 = Never, 2 = Rarely, 3 = Sometimes, 4 = Often, 5 = Always**

No	Statement	1	2	3	4	5
1	I prioritize using cloth towels/gowns over disposable paper ones for patients/procedures where clinically appropriate.					
2	I minimize the use of single-use plastic drapes and coverings during deliveries/minor procedures.					
3	I ensure all clean recyclable packaging from medical supplies (e.g., paper wraps, cardboard boxes) is properly recycled.					
4	I power down electronic fetal monitors or other equipment immediately after the patient is discharged/equipment is not needed.					
5	I opt for saline flushes in reusable vials rather than single-use pre-filled syringes when possible and policy allows.					
6	I carefully manage the amount of supplies opened for each delivery/procedure to avoid unnecessary waste.					
7	I check expiration dates frequently to minimize the disposal of expired medications and supplies.					
8	I use environmentally friendly, green cleaning products within the maternity unit if they are provided.					
9	I turn off lights/equipment when they are not in use to conserve energy.					
10	I minimize paper usage (e.g., opting for digital records over printouts).					
11	I actively participate in the unit's recycling programs.					
12	I choose reusable equipment/materials over single-use alternatives when clinically safe and available.					
13	I ensure proper segregation of general, clinical, and hazardous waste.					
14	I use water conservation practices during medical procedures or cleaning.					
15	I consider the environmental impact when selecting medical supplies for procedures.					
16	I report inefficiencies in waste management or energy use in my unit.					

17	I manage supply "prep" carefully to avoid opening unnecessary items that will then be wasted.					

Section S4: Barriers to Sustainable maternity Nursing Practice

Use a Likert scale: 1 = Not a Barrier, 2 = Minor Barrier, 3 = Moderate Barrier, 4 = Major Barrier, 5 = Significant Barrier

No	Statement	1	2	3	4	5
1	Time constraints during emergencies prevent focusing on waste segregation or energy saving.					
2	The layout of the L&D rooms makes proper waste segregation bins difficult to access.					
3	A lack of supportive management or clear policies on sustainability from hospital leadership.					
4	Concerns that "green" products or reusable items may compromise infection control standards.					
5	Insufficient supply of sustainable alternatives to current single-use products.					
6	Lack of consistency in sustainability efforts among all L&D staff members (nurses, doctors, technicians).					

Section S5: Barriers and Institutional Support

Use a Likert scale: 1 = Strongly Disagree, 2 = Disagree, 3 = Neutral, 4 = Agree, 5 = Strongly Agree

No	Statement	1	2	3	4	5
1	There is a lack of clear guidance from management on sustainable practices in my unit.					
2	Time constraints during busy shifts make it difficult to focus on waste segregation or energy saving.					
3	The hospital's current procurement policies do not prioritize environmentally friendly products.					
4	There is a "buy-in" problem among my colleagues; many are not interested in sustainability efforts.					
5	I have received formal training on waste management specific to the operating room or delivery ward.					
6	The cost of sustainable alternatives is perceived as a major barrier by the administration.					

Section S6: Eco-conscious Advocacy and Attitudes

Use a Likert scale: **1 = Strongly Disagree, 2 = Disagree, 3 = Neutral, 4 = Agree, 5 = Strongly Agree**

No	Statement	1	2	3	4	5
1	I actively encourage my colleagues to adopt more sustainable practices (e.g., proper recycling, turning off equipment).					
2	I discuss the importance of environmental health with patients and their families when appropriate.					
3	I believe nurses have a professional obligation to advocate for environmental protection within their healthcare facility.					
4	I have participated in a hospital-wide initiative or committee related to "greening" the hospital.					

5	I would be willing to dedicate my own time to lead a sustainability project in the maternity unit.					
6	I believe that being "green" improves patient outcomes and safety in the long run.					
7	I feel empowered to suggest process improvements that reduce waste in the maternity unit.					
8	I believe that nurses in the maternity unit are key players in achieving hospital sustainability goals.					
9	Advocating for a healthy environment is a natural extension of my nursing role to protect public health.					
10	I discuss environmentally friendly choices regarding newborn care (e.g., product selection) with new parents.					

Section S7: <u>Specific Procedural Concerns (Anesthesia, Instruments, Waste):-</u>

<u>Use a Likert scale: 1 = Strongly Disagree, 2 = Disagree, 3 = Neutral, 4 = Agree, 5 = Strongly Agree</u>

No	Statement	1	2	3	4	5
1	The environmental impact of anesthetic gases (e.g., nitrous oxide) used for pain relief in labor is a concern for me.					
2	Our unit effectively separates "red bag" regulated medical waste from standard general waste to reduce costs and environmental impact.					
3	I am aware of the specific disposal method for single-use fetal monitoring straps/sensors.					
4	We use steam sterilization (autoclave) of instruments more often than chemical sterilization to reduce chemical waste					
5	I believe single-use plastic drapes used during C-sections or deliveries could be replaced with sterilizable cloth ones.					

Section S8: <u>Specific Procedures and Material Management):-</u>

<u>Use a Likert scale: 1 = Strongly Disagree, 2 = Disagree, 3 = Neutral, 4 = Agree, 5 = Strongly Agree</u>

No	Statement	1	2	3	4	5
1	I am aware of the environmental burden associated with single-use plastics used in delivery kits/trays.					
2	Our unit over-packs procedure trays, leading to unnecessary waste of supplies.					
3	I feel our unit has effective systems for returning unopened, unused sterile supplies to stock.					
4	I use the minimum amount of antiseptic solution/disinfectant required for procedures to avoid chemical waste.					
5	I am comfortable suggesting the use of reusable cloth items (e.g., surgical gowns/drapes, baby blankets) over disposables when appropriate.					
6	Anesthesia gases used during deliveries have a significant environmental impact.					
7	I advocate for the use of less environmentally harmful pain management options during labor (where clinically safe).					

Section S9: <u>Energy and Water Consumption in the Unit):-</u>

<u>Use a Likert scale: 1 = Strongly Disagree, 2 = Disagree, 3 = Neutral, 4 = Agree, 5 = Strongly Agree</u>

No	Statement	1	2	3	4	5
1	I ensure non-critical monitors and medical devices are powered off when not in use. (Frequency: 1-5).					
2	Our maternity unit's current lighting system is energy efficient (e.g., LED). (Agreement: 1-5)					
3	I take steps to minimize running water during bathing, cleaning, or other procedures. (Frequency: 1-5)					
4	The temperature in the maternity unit is often too high, leading to wasted energy. (Agreement: 1-5)					
5	I am aware of the specific energy consumption 'hotspots' in the maternity ward (e.g., incubators, warmers). (Agreement: 1-5)					
